# Circulating tumour cells in breast cancer

**DOI:** 10.3332/ecancer.2013.352

**Published:** 2013-09-19

**Authors:** Natalia Krawczyk, Malgorzata Banys, Andreas Hartkopf, Carsten Hagenbeck, Carola Melcher, Tanja Fehm

**Affiliations:** 1 Department of Obstetrics and Gynecology, University of Duesseldorf, Moorenstr. 5, 40225 Duesseldorf, Germany; 2 Department of Obstetrics and Gynecology, University of Tuebingen, Calwerstr. 7, 72076 Tuebingen, Germany

**Keywords:** breast cancer, disseminated tumour cell, circulating tumour cell, prognosis, biomarkers

## Abstract

Evaluation of isolated tumour cells in bone marrow (BM) and peripheral blood has become a major focus of translational cancer research. The presence of disseminated tumour cells in BM is a common phenomenon observed in 30–40% of primary breast cancer patients and independently predicts reduced clinical outcome. The detection of circulating tumour cells (CTCs) in blood might become a desired alternative to the invasive and painful BM biopsy. Recent clinical trials confirmed the feasibility of CTC detection as a robust and reproducible parameter for prognostication in both adjuvant and metastatic setting. The characterisation of CTCs might become an important biomarker for therapy monitoring and help to identify specific targets for novel therapeutic strategies.

## Introduction

Distant metastasis is the main cause of tumour-related death, but the occult spread of isolated tumour cells (ITCs) in the earliest stage of breast cancer remains undetected by conventional imaging technologies. ITCs in secondary sites, such as blood and bone marrow (BM), are assumed to be precursors of (micro)metastatic disease. The phenomenon of haematogenous dissemination in the metastatic cascade was recognised by several researchers in 19th century [1]. Therefore, detection and characterisation of these cells have become a major focus of translational cancer research. Sensitive assays enable reproducible evaluation of disseminated tumour cells (DTCs) and circulating tumour cells (CTCs) at the single-cell stage.

As demonstrated by a large pooled analysis, the presence of DTCs in BM at the time of diagnosis is associated with reduced survival [2]. In recent years, numerous research groups have endeavoured to replace the invasive and painful BM biopsy with a simple blood test. In the following review, we will discuss the current clinical value of CTCs in the early and advanced breast cancer.

## Detection methods

The low frequency of ITCs, estimated at one tumour cell/10^7^–10^8^ blood cells in patients with advanced cancer, explains the need for extremely sensitive detection assays and tumour cell enrichment [3, 4]. Currently, antibody-based and molecular methods are the main techniques for CTC detection.

### Tumour cell enrichment

Three main methods of tumour cell enrichment are currently in use: (a) density gradient centrifugation leads to the separation of mononuclear cells from other blood cells; (b) positive selection leads to the enrichment of CTCs through the use of an antibody targeted against, e.g., cytokeratins (CKs) or epithelial cell adhesion molecule (EpCAM); or (c) negative selection, where the antibody is targeted against a leucocyte antigen (e.g., CD45).

### Antibody-based CTC detection

The majority of translational research trials use antibodies against markers absent from other blood cells; due to the lack of breast cancer-specific antigens, commonly used markers are of epithelial origin (e.g., EpCAM and CKs) [5]. CTCs are then identified by the staining pattern and morphological criteria (consensus recommendations for DTC detection provide a list of phenotypic features that allow a reproducible differentiation between tumour and blood cells) [4]. Antibody-based techniques allow for a direct quantification of detected tumour cells.

### Molecular methods

Molecular methods are mainly based on reverse transcription polymerase chain reaction (RT-PCR) amplification of epithelial or tissue-specific messenger ribonucleic acid (mRNA). Various markers have been established for molecular CTC detection including EpCAM, CKs, and mammaglobin [6]. A limiting factor for RT-PCR-based detection is the illegitimate low-level transcription of targeted mRNA in normal cells and presence of pseudogenes [14]. Therefore, quantitative real-time RT-PCR frequently uses a cut-off value to differentiate between positive and negative findings.

An additional valuable tool for the evaluation of various markers and further characterisation of CTCs with regard to predictive markers, such as human epidermal growth receptor 2 (HER2) or hormone receptor status, is represented by semiquantitative multiplex PCR [7].

### Commercially available assays

A variety of commercially available standardised assays for CTC detection has been developed over the last few years. The most commonly used test is the semiautomated, antibody-based, US Food and Drug Administration (FDA)-cleared CellSearch System (Veridex, Warren, New Jersey, United States). This quantitative assay is based on immunofluorescence [8, 9]. After the CTC enrichment by immunomagnetic beads linked with anti-EpCAM antibodies, tumour cells are identified by CK positivity, positive nuclear staining, and CD45 negativity.

One of the most widely used molecular tools for CTC detection is the AdnaTest BreastCancer (AdnaGen AG, Langenhagen, Germany). This tool assay is based on semiquantitative RT-PCR. CTCs are enriched by immunomagnetic beads linked with anti-mucin 1 (MUC1) and anti-EpCAM antibodies and mRNA of three markers gastrointestinal tumour-associated antigen [(GA 733.2), EpCAM, and HER2] is amplified by a multiplex PCR [7, 10, 11]. Both tests have been compared in our previous study and a concordance rate of 70–90% has been reported.

## Detection of CTC in early breast cancer

Despite its complete surgical extirpation, breast cancer has the ability to recur years after primary diagnosis. As this might happen even in patients without lymph node involvement, occult haematogenous spread of the tumour seems to occur long before the primary tumour becomes clinically detectable. After primary tumour extirpation, tumour cell dissemination leads to minimal residual disease (MRD) and consequently, the aim of successful adjuvant treatment must be its complete eradication. A considerable amount of recent literature on the presence of DTCs in the BM of primary breast cancer patients strongly supports this hypothesis. DTC detection at the time point of diagnosis as well as the detection of persistent DTCs during follow-up is associated with an impaired prognosis and DTCs are currently regarded as an important surrogate of MRD [2, 12–25].

DTC detection is an invasive procedure and associated with increased morbidity. Thus, recent attempts have been made to implement less painful methods that detect CTCs in the peripheral blood (PB) of primary breast cancer patients. However, reports on the prevalence of CTC detection and their prognostic impact are incoherent, which is mainly due to different detection methods and varying cut-off values for positive samples [26–38]. Available literature on the prevalence and prognostic relevance of CTCs, detected in early breast cancer patients, is summarised in Table 1.

**Figure 1.**
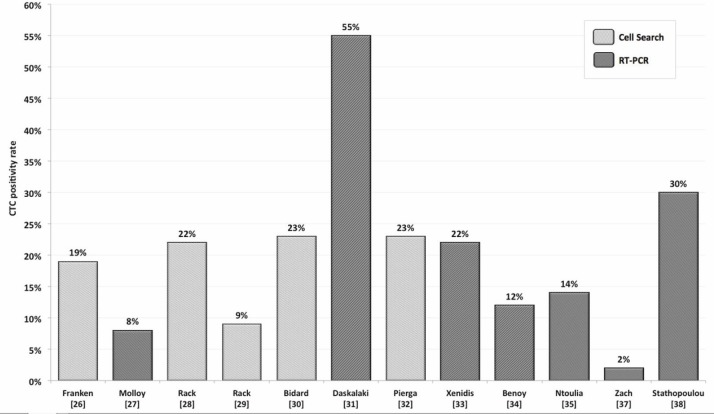
CTC positivity rates depending on detection method reported by several authors.

In the adjuvant situation, the translational research program of the German SUCCESS-trials prospectively investigated the clinical relevance of CTCs in a large number of primary breast cancer patients. Using the CellSearch System (Veridex), 22% of the patients presented with ≥1 CTC/7.5-ml PB at primary diagnosis, and CTC detection before taxane-based chemotherapy was an independent predictor of disease-free survival (DFS; HR 1.88) and overall survival (OS; HR 1.91) [28]. Similar results on the prognostic impact of CTCs were reported previously by smaller studies using the CellSearch System or RT-PCR-based techniques for CTC detection (Table 1 and Figure 1) [26, 27, 30–38].

In patients receiving neoadjuvant therapy, monitoring of the primary tumour response allows to evaluate the success of systemic treatment. However, patients might suffer from a relapse despite pathological complete remission. This indicates that systemic response to treatment is independent from the local treatment response of the primary tumour. Accordingly, monitoring of MRD by CTC/DTC detection during neoadjuvant treatment offers the possibility to gain better insights into the influence of systemic treatment on tumour cell dissemination and should also help to optimise treatment strategies.

Interestingly, most of the studies indicate that CTC/DTC detection after neoadjuvant systemic treatment is independent from the primary tumour response and not associated to any clinicopathological characteristics of breast cancer [32, 39–42]. Moreover, in the German GeparQuattro and GeparQuinto trials, the prevalence of CTC detection decreased under neoadjuvant chemotherapy [43, 44]. Mathiesen *et al * [40] who evaluated the CTC/DTC status before and after neoadjuvant treatment, also found that the number of CTC/DTC-positive patients was decreasing. Whereas, in accordance with other data, DTC detection after neoadjuvant treatment was prognostic of survival, the CTC status had no impact on prognosis. The recently published BEVERLY-2 study also found no prognostic relevance of CTC detection after neoadjuvant treatment [45]. These results seemed to be caused by an increased sensitivity of CTC detection methods as compared with DTC detection. By contrast, Bidard *et al* [30] and Pierga *et al* [32] observed a prognostic impact of CTCs detected before and after therapy for early relapse. Similarly, Rack *et al *presented data of persistent CTC from the adjuvant SUCCESS trials. The persistence of ≥1 CTC after chemotherapy was associated with decreased DFS and the persistence of ≥5 CTCs was associated with decreased OS [29].

### Characterisation of CTCs

ITCs in PB and BM of breast cancer patients are regarded as a surrogate marker for MRD. Therefore, beyond local therapy of primary tumour and lymph node metastases, the eradication of ITC has become a desirable goal of breast cancer treatment. Thus, further characterisation of CTCs, as potential targets for adjuvant therapies, is gaining in importance. However, the choice of systemic treatment is presently based on expression profile of the primary tumour rather than on that of MRD [39].

Phenotypic differences between primary tumour and ITCs in blood and BM have been reported in [7, 46–49]. This phenomenon might be of clinical relevance for treatment decisions concerning endocrine or targeted therapy. Pestrin *et al* [50] showed a considerable discrepancy in HER2 status in 66 patients with metastatic breast cancer; 29% of these patients presented with HER2-positive CTCs despite HER2-negative primary tumour, whereas 42% of patients showed HER2-negative CTCs and HER2-positive primary lesion. In our previous trial, we found HER2-positive DTCs persisting in patients with HER2-negative primary tumour after the completion of adjuvant therapy [48]. Moreover, according to the molecular analyses HER2 gene amplification can be acquired during disease progression [51]. Since HER2-targeted therapy is intended only for patients with HER2-positive primary tumour, HER2-positive MRD in patients with HER2-negative primary lesion remains untreated and may subsequently cause metastasis.

A number of trials have investigated the influence of HER2-targeted therapy on CTCs/DTCs. Bernhard *et al* [52] transferred autologous HER2-specific T-lymphocytes to a patient with HER2-positive metastatic breast cancer. This experimental treatment was able to eliminate HER2-positive DTCs from the BM, but did not influence the growth of solid metastases. In an interventional study by Rack *et al*, ten primary breast cancer patients with persistent HER2-positive DTCs received trastuzumab therapy for 12 months. DTC status was then revaluated by follow-up BM biopsies at regular time intervals [53]. HER2-positive DTCs were eradicated in all the patients. However, clinical significance of MRD elimination remains yet unclear. Two randomised clinical trials, DETECT III and TREAT CTC, have been initiated recently to evaluate whether patients with persistent ITCs benefit from HER2-targeted therapy based on HER2 status of their CTCs [54, 55].

**Figure 2.**
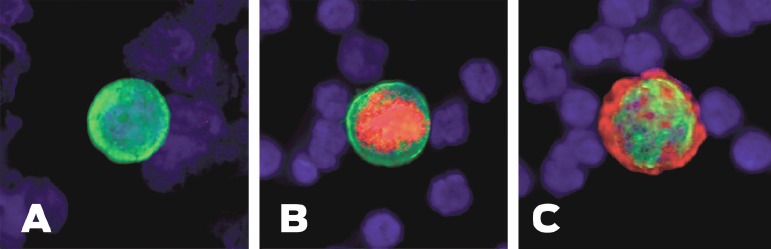
Heterogeneity of ITCs shown by immunofluorescence. (A) Cytokeratin positive ITC. (B) Cytokeratin and ER positive ITC. (C) Cytokeratin and HER2-positive ITC.

Phenotypic discrepancies between primary tumour and MRD have been reported with regard to hormone receptor status as well. A number of studies have shown that CTCs/DTCs are generally hormone receptor negative in spite of hormone receptor positive primary tumour [47, 56, 57]. Previously, we evaluated ER status of DTCs in 107 primary breast cancer patients. Only 12 of 88 patients (14%) with ER-positive primary tumour presented with ER-positive DTCs in BM while the majority (86%) had ER-negative DTCs [49]. The discrepancy in ER status between primary tumour and MRD may explain the failure of endocrine therapy in a subset of ER-positive patients. Figure 2 shows the heterogeneity of ITCs.

## CTC detection in metastatic breast cancer

According to a number of studies, 40–80% of metastatic breast cancer patients present with CTCs in PB. Above the cut-off value of ≥5 CTCs/7.5-ml PB, a patient is considered CTC positive [8, 9]. The most common tool used for CTC detection in advanced breast cancer is the FDA-approved CellSearch System.

### Prognostic value of CTCs in advanced breast cancer

Prognostic significance of CTCs in metastatic breast cancer has been demonstrated in a number of clinical trials to date (Table 2). The first study to investigate the impact of CTCs on clinical outcome and select an optimal cut-off point for CTC count in this collective of patients was published in [8]. In this multicentre prospective trial, metastatic breast cancer patients with at least five CTCs/7.5-ml PB had significantly shorter progression-free survival (PFS) and OS compared to patients with <5 CTCs/7.5 ml (median PFS: 2.7 versus seven months and OS: 10.1 versus 18 months) [8]. Thus, the cut-off of ≥5 CTCs/7.5-ml PB is used to distinguish between patients with good or poor clinical outcome.

In [58], CTC levels in the same patients cohort were subsequently evaluated at additional time points: before the start of treatment, at 3–5, 6–8, 9–15, and 15–20 weeks of follow-up. Interestingly, the prognostic power of the cut-off value of ≥5 CTCs/7.5-ml PB with regard to PFS and OS remained unchanged. Moreover, the authors observed dynamic changes in CTCs during therapy; the decrease in CTC levels from at least 5/7.5-ml PB to <5/7.5-ml PB during the treatment was associated with a better PFS and OS compared with persistent high CTC counts.

Furthermore, the prognostic value of CTC detection has been recently investigated with regards to molecular subtypes of breast cancer. According to [59], CTCs have no predictive impact on the survival in patient with metastatic HER2-positive breast cancer treated with HER2-targeted therapy, in contrast to all other subtypes of breast cancer. This effect might be due to selective effectiveness of HER2-directed therapy against CTCs. These results are concordant with those reported in [60]; in their prospective trial, the authors showed stronger decrease in CTC counts in patients who received targeted therapy (trastuzumab or bevacizumab) in addition to first-line chemotherapy. In contrast, a large prospective multicentre study in [61] reported strong independent prognostic impact of CTCs in metastatic breast cancer patients independent of molecular subtype.

Clinical significance of high CTC levels in patients with metastatic breast cancer irrespective of location or number of metastatic sites and hormone receptor status or HER2 status of tumour has been confirmed in a large trial in [62] (median PFS 12.0 versus seven months for patients with CTCs <5 and ≥5, respectively; *p* < 0.001). In this retrospective study of 235 metastatic breast cancer patients, the authors investigated a predictive value of CTC levels for efficiency of the different treatment regimens. Interestingly, patients with high (≥5) baseline CTC counts showed only marginal survival benefit from first-line endocrine treatment despite positive hormone receptor status of primary tumour or metastatic lesion. Phenotypic discrepancies between primary tumours and ITCs might be one of the possible factors limiting endocrine therapy in this collective of patients. Therefore, alternative treatment approaches (e.g., antineoangin genetic or targeted therapy) should be evaluated for this population.

### Therapy monitoring

Therapy response in metastatic breast cancer patients is presently being assessed by clinical examination, radiological imaging, and levels of tumour markers in PB. These approaches might be insufficient, and thus new reliable tools are necessary to serve as surrogate markers for the efficiency of treatment. Since changes in CTC counts seem to reflect therapy responses as early as after the first cycle of chemotherapy, CTC dynamics in patients with advanced breast cancer may serve as a new therapy monitoring tool.

Budd *et al* [63] in their prospective multicentre trial compared the predictive impact of CTC detection to standard imaging with regard to treatment response in metastatic breast cancer patients [63]. The median OS of patients with therapy response assessed by radiological imaging and ≥5 CTCs in PB was significantly shorter than that of patients with therapy response by radiological assessment and <5 CTCs (15.3 versus 26.9 months; *p* = 0.0389). Moreover, patients with radiologic progression and <5 CTCs in PB had significantly longer OS comparing patients with radiological progression and ≥5 CTCs in PB (19.9 versus 6.4 months; *p* = 0.0039).

Whether patients with advanced breast cancer benefit from treatment decisions based on CTC dynamics during therapy is yet unclear. In an attempt to answer this question, the SWOG S0500 trial has been initiated by the Southwest Oncology Group (NCT00382018). The aim of this randomised phase-III study is to investigate whether patients with advanced breast cancer and persistently high levels of CTC (≥5/7.5 ml of blood) after three weeks of first-line chemotherapy benefit from switching to an alternative chemotherapy regimen versus waiting for clinical evidence of disease progression before the initiation of new treatment [64]. The recruitment was finished in 2012.

### CTC assessment as a real-time ‘liquid biopsy’ in metastatic breast cancer

Several studies have reported the phenotypic and genotypic discrepancies between primary tumours, ITC in secondary sites and solid metastases [7, 46–49]. Changes in expression profile of breast cancer can be acquired in the course of the disease [51]. Therefore, the characteristics of the primary tumour, especially with regard to hormone receptor status and HER2 status may not reflect the phenotype of metastases. Since repeated tissue sampling of solid metastatic lesions is not feasible, the assessment of tumour status on CTCs by a simple blood draw as a ‘liquid biopsy’ might represent an adequate alternative [65].

We previously evaluated HER2 status of CTCs in 254 metastatic breast cancer patients at the time of first diagnosis or disease progression using the CellSearch System. In this prospective multicentre trial, we found HER2-positive CTCs in 25 of the 78 patients (32%) with HER2-negative primary tumour [66]. The clinical value of these findings is being addressed in the prospective randomised DETECT III trial [54], which started recruitment in February 2012. This study investigates the efficacy of anti-HER2 treatment with Lapatinib in advanced breast cancer patients with HER2-positive CTCs, but HER2-negative primary tumour. Similarly, recently initiated TREAT CTC trial should evaluate whether breast cancer patients in adjuvant setting benefit from anti-HER2 therapy based on CTC persistence [55].

## Conclusions

The presence of CTCs in PB of breast cancer patients is associated with impaired clinical outcome in both primary and metastatic settings. CTC detection might lead to the establishment of new treatment strategies in early and advanced breast cancer. However, beyond the mere enumeration, further characterisation of CTCs is gaining in importance. New strategies of targeted treatment based on expression profiles of CTCs are being currently investigated in clinical trials, since the phenotype of CTCs may differ from that of primary tumour.

## Figures and Tables

**Table 1.** Prognostic relevance of CTC detection in primary breast cancer patients.

Author	Year	*N*	Method	Positivity rate (%)	Follow-up (months)	Prognostic relevance
Franken *et al *[26]	2012	404	CellSearch	19^4^	48	DFS^5^, BCSS^6^
Molloy *et al *[27]	2011	733	RT-PCR	8	91	DFS, BCSS
Rack *et al *[28]	2010	2,026	CellSearch	22^1^	35	DFS, OS
Rack *et al *[29]	2010	1,489	CellSearch	9^4^	32	DFS^2^, OS^1^
Bidard *et al *[30]	2010	115	CellSearch	23	36	DFS, OS
Daskalaki *et al *[31]	2009	165	RT-PCR	55^1^, 52^2^	59	OS^1^
Pierga *et al *[32]	2008	118	CellSearch	23^1^, 17^2^	18	DFS^3^
Xenidis *et al *[33]	2006	167	RT-PCR	22	32	DFS, OS
Benoy *et al *[34]	2006	116	RT-PCR	12–14	26	None
Ntoulia *et al *[35]	2006	101	RT-PCR	14	24	DFS
Nieto *et al *[36]	2004	242	ICC	7	84	DFS, OS
Zach *et al *[37]	2002	218	RT-PCR	2	>12	DFS
Stathopoulou *et al *[38]	2002	148	RT-PCR	30	28	DFS, OS

1 Before chemotherapy.

2 After chemotherapy.

3 Combined positivity before and/or after neoadjuvant chemotherapy.

4 At least one CTC.

5 Multivariate analysis.

6 Univariate analysis.

ICC: Immunocytochemistry.

**Table 2.** Prognostic relevance of CTC detection in metastatic breast cancer patients.

Author	Year	Number of patients	Method	Positivity rate (%)	Prognostic relevance
Wallwiener *et al *[61]	2013	486	CellSearch	42	PFS, OS
Giordano *et al *[59]	2012	517	CellSearch	40^1^	PFS, OS
Pierga *et al *[60]	2012	267	CellSearch	44^1^	PFS, OS
Müller *et al *[67]	2012	254	CellSearch AdnaTest	CSS: 50^1^ AT: 40	CellSearch: OS AdnaTest: none
Giuliano *et al *[62]	2011	235	CellSearch	40^1^	PFS, OS
Reinholz *et al *[68]	2011	86	RT-PCR	56–75^2^ 23–38^3^	OS^2^ None^3^
Nakamura *et al *[69]	2010	107	CellSearch	37^1^	PFS
Liu *et al *[70]	2009	74	CellSearch	n.s.	PFS
Tewes *et al *[71]	2009	42	AdnaTest	52	OS
Bidard *et al *[72]	2008	37	ICC	41	OS
Nole *et al *[73]	2008	80	CellSearch	61	PFS
Hayes *et al *[58]	2006	177	CellSearch	54	PFS, OS^4^
Budd *et al *[63]	2006	138	CellSearch	43	OS
Benoy *et al *[34]	2006	32	RT-PCR	25–40	None
Cristofanilli *et al *[8]	2004	177	CellSearch	49	PFS, OS

1 ≥5 CTCs.

2 CK19 mRNA.

3 Mammaglobin mRNA.

4 At any time during palliative treatment.

n.s.: not specified.
